# Abnormal branch of right pulmonary artery (A7): a case report and literature review

**DOI:** 10.1186/s40792-016-0141-x

**Published:** 2016-02-15

**Authors:** Maiko Atari, Yuki Nakajima, Mitsuro Fukuhara, Yoshihito Iijima, Hiroyasu Kinoshita, Hirohiko Akiyama, Yoshihiro Minamiya, Hidetaka Uramoto

**Affiliations:** Department of Thoracic Surgery, Saitama Cancer Center, 780 Komuro, Ina, Kita-adachi-gun, Saitama, 362-0806 Japan; Department of Thoracic Surgery, Akita University Graduate School of Medicine, Akita, Japan

**Keywords:** Pulmonary artery, Abnormal branch, 3D-CT

## Abstract

In thoracic surgery, anatomic variations of pulmonary artery increase the risks for vessel injury and critical mistakes during pulmonary artery resection. We report a case of lung cancer with an extremely rare branch, a mediastinal A7 pulmonary artery. Some case reports of the mediastinal pulmonary artery exist until now. However, to the best of our knowledge, this is the first case of a medial basal segmental artery (from the following, it is referred to as A7) branching directly from main pulmonary artery in the literature. Therefore, there is no report that showed three-dimensional computed tomography (3D-CT) and operative findings. So, these information is very useful for thoracic surgeon. A 67-year-old man was admitted to our hospital in order to undergo operation for the treatment of lung cancer. We detected the anomalies preoperatively by 3D-CT. The 3D-CT shows the A7 pulmonary artery branches from the right main pulmonary artery directly. According to previous literature, the cases of a single branch from main pulmonary artery to lower lobe are only five cases. And, the only two of them are right side including our case. In spite of an extremely rare case, we were able to successfully perform a right middle lobectomy because the information obtained from the 3D-CT findings was sufficiently understood preoperatively.

## Background

Branching of the pulmonary artery, pulmonary vein and bronchus vary among individuals [1], and abnormal blood vessel and vascular branch are observed in rare cases. Sharing of this information with the team is important for the performance of safe surgery. Low-invasive surgery, such as video-assisted thoracic surgery (VATS) and segmentectomy, is increasingly performed, and preoperative confirmation of the vascular branching is important in this surgery. Computed tomography (CT) and reconstructed vascular images from 3D-CT angiography are useful for this purpose [2, 3]. Herein, we summarize the key points during surgery and review the literature on similar cases.

## Case presentation

**Fig. 1:**
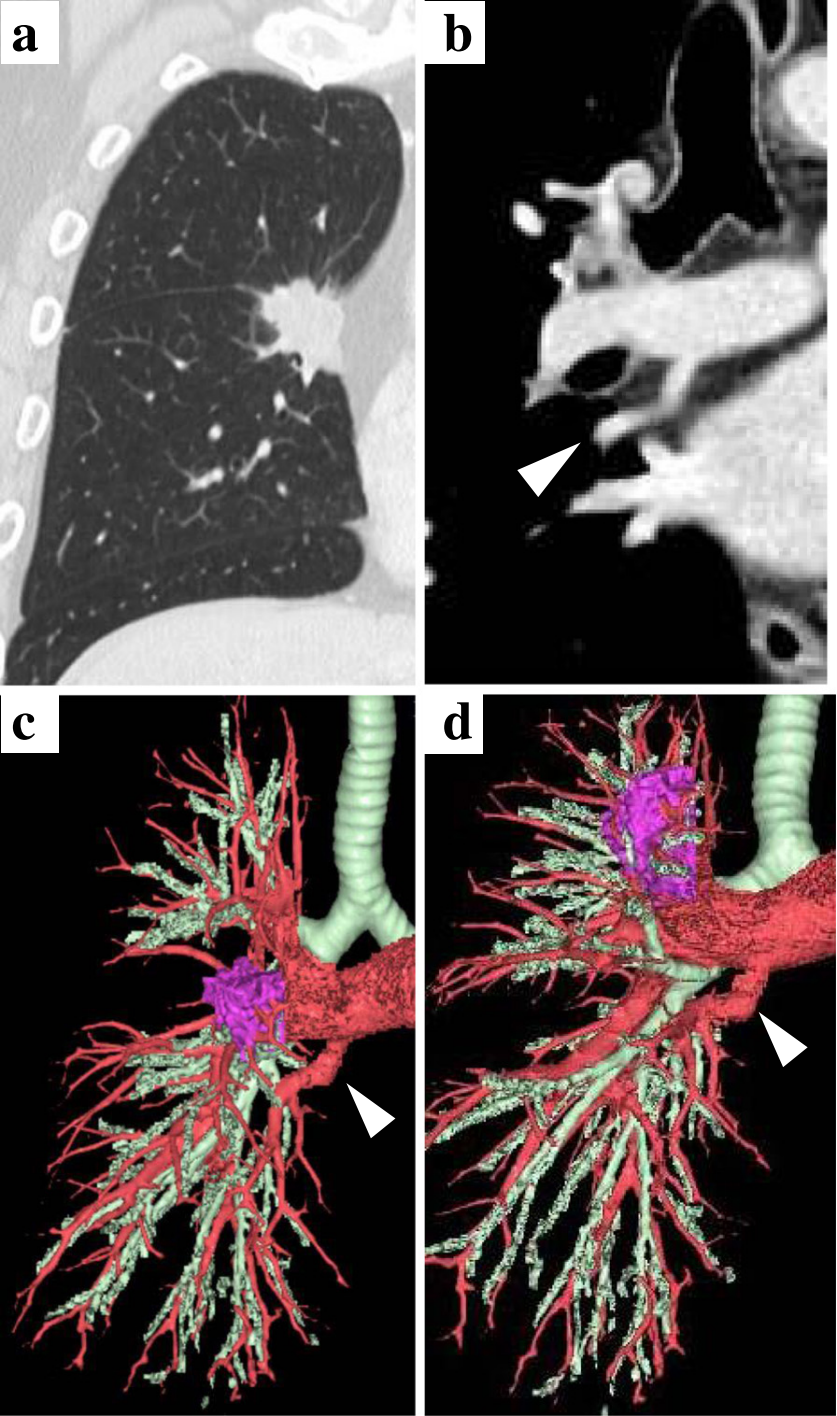
The tumor exists in the right middle lobe and is suspected the infiltration into the mediastinal fat (T4) in the preoperative chest CT (**a**). And, the coronal CT shows abnormal A7 (◁) branching from right main pulmonary artery (**b**). The three-dimensional computed tomography is the similar findings (**c**). If we look from the bottom a little, branching from main PA was easier to understand (**d**)

The patient was a 67-year-old man in whom an abnormal shadow was detected on plain chest radiography in medical checkup in February 2015. Lung cancer was detected on bronchoscopy and was diagnosed as cT4 (mediastinal fat invasion) N0M0 stage IIIA. There was no particular medical history other than treatment of hepatitis C and psoriasis. The patient had smoked 10 cigarettes per day for 40 years but had stopped smoking 1 year earlier. In blood tests, there was no elevation of tumor markers and only mild liver dysfunction (aspartate aminotransferase: 33 IU/L, alanine aminotransferase: 28 IU/L). Branching of A7 from the right main pulmonary artery was noted on preoperative CT and 3D angiography (Fig. 1). There was no abnormal branching of the bronchus on bronchoscopy.

Right middle lobectomy, combined partial resection of the right upper lobe, and mediastinal lymph node dissection were performed by VATS. A7 branching from the right main pulmonary artery was observed during surgery, as detected on CT and 3D angiography (Fig. 1), and attention to the A7 was required during interlobular formation between the upper and middle lobes. Surgery was completed without a trouble. The chest drain was removed on postoperative day 2, and the patient was discharged on day 7. The final pathological diagnosis was lung squamous cell carcinoma, pT2a (PL3) N0M0 pStage IB. As of 7 months after surgery, there has been no recurrence.

### Discussion

Low-invasive procedures such as VATS and segmentectomy are becoming increasingly used as the surgical indication has expanded for lung cancer discovered in an early stage. Preoperative confirmation of the vascular variation is particularly important for safe performance of these surgeries. Since variation of veins in the lower lobe is diverse compared to that in the upper lobe, the distribution of veins cannot be used as an index to identify arteries in segmentectomy of the lower lobe, and identification of the branching of arteries in the segment is important [1]. Regarding the A7 branching pattern, A7 and A8 branch from the same trunk in 60 % of cases, A7 independently branches from the basal trunk in 34 %, and A7 is absent in 6 % [1]. Thus, A7 branching from the pulmonary artery trunk is very rare. In comparison of the bilateral sides, the right pulmonary artery is longer and thicker than the left pulmonary artery, but the branching pattern of the left pulmonary artery is more diverse. The pulmonary arteries develop from the bilateral sixth branchial arch arteries. The abnormal branch is thought to occur during remodeling of these arteries, but the detailed reasons are not understood.

Most cases with abnormal branch of the pulmonary artery have accompanying abnormal bronchial bifurcation, and the incidence of such cases is higher in the upper lobe. Direct branching of the lower pulmonary artery from the thoracic aorta is occasionally observed, such as that in cases of pulmonary sequestration and anomalous arising of the basal segmental artery. However, very few cases with a mediastinal basal pulmonary artery, in which the pulmonary artery directly branches from the pulmonary artery trunk to the lower lobe, have been described. We searched similar cases on PubMed, a service of the National Library of Medicine (http://www.ncbi.nlm.nih.gov/pubmed). The search focused on the key words “mediastinal pulmonary artery,” “abnormal vessel of lung,” and “abnormality of the pulmonary artery.” The search identified only Japanese reported cases. Only 4 right-side cases, including our patient, and 12 left-side cases have been reported in Japan (Table 1, [2, 4–12]). The lower pulmonary artery independently branched from the main pulmonary artery in 5 of these cases, and only in 2 right-side cases, including our patient. Abnormal distribution of the lower pulmonary artery combined with the middle or lingular pulmonary artery was observed in 7 cases, and the middle lobectomy was performed in only 2 cases. There has been a recent increase in the number of such case reports, which may be due to the increased use of 3D-CT angiography. The identification rate of abnormal branches by preoperative 3D-CT is 98 %, compared to intraoperative operative findings [3]. Certainly, Anomaly in vessels was pointed out in all of 5 cases undergoing preoperative 3D-CT evaluation. Preoperative evaluation be 3D-CT was not performed in 4 cases whose vessel anomaly was not recognized. 3D-CT is capable of vessel construction even without a contrast agent, so it would be ideal to take in the routine preoperatively.

**Table 1:** Reports of mediastinal basal pulmonary artery: review of literature

	Right			Left		
		*n*	Rate		*n*	Rate (%)
Procedure	RUL	0	0	LUL	5	42
	RML	2	50	LLL	6	50
	RLL	2	50	Unknown	1	8
Pattern of branch from main PA	Basal (single)	1	25	Basal (single)	3	25
	Basal (multiple)	2	50	Basal (multiple)	3	25
	Complex with middle lobe artery	1	25	Complex with lingular lobe artery	6	50
Preoperative diagnosis	Obtained	4	100	Obtained	8	66
	Not obtained	0	0	Not obtained	4	33

#### Operative findings and notes in surgery

**Fig. 2:**
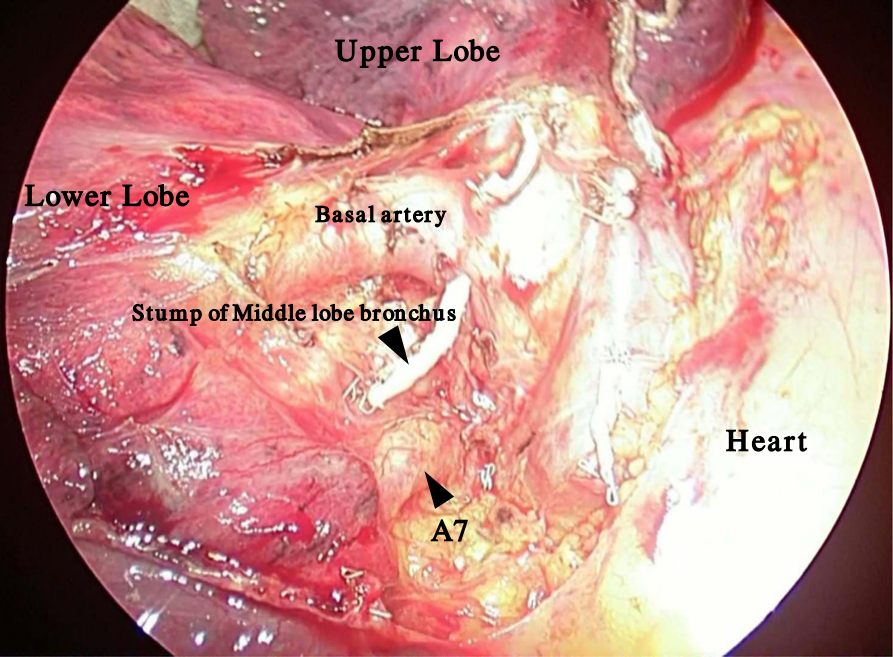
Operative findings. We took care of interlobular formation in RML, because A7 passed through the region posterior to the superior pulmonary vein and was also distributed anterior to the inferior pulmonary vein

In our patient, A7 passed through the region posterior to the superior pulmonary vein and was also distributed anterior to the inferior pulmonary vein, while projecting a thin branch toward the posterior region of the inferior pulmonary vein. A pulmonary artery is not normally present in the posterior region of the inferior pulmonary vein, and thus resection of the lower lobe and segmentectomy in this patient would have had a risk of injuring the blood vessel if the A7 branch had not been identified beforehand. Required reconstruction of a mediastinal basal pulmonary artery (A8+9), which was regarded as a mediastinal lingular artery and erroneously transected, has been reported in a patient treated with the left upper lobectomy [13]. Thus, evaluation of the vascular distribution on preoperative CT in combination with 3D-CT angiography is useful to prevent unexpected hemorrhage during surgery and transection of an artery outside the range of resection (Fig. 2).

## Conclusions

Since the surgery was the right middle lobectomy, careful attention to this A7 branch was required during interlobular formation. Vascular dissection should be performed carefully in the right lower lobectomy and segmentectomy in patients with this pulmonary arterial distribution. Preoperative determination of the vascular distribution using CT and 3D-CT angiography is particularly important for safe VATS and segmentectomy and will continue to be important because of the recent increase in this type of surgery.

## Consent

Consent was obtained from the patient for the publication of this case report.

## Abbreviations

CT: computed tomography
3D-CT: three-dimensional computed tomography
VATS: video-assisted thoracoscopic surgery
